# Case report: First-in-human combined low-dose whole-heart irradiation and high-dose stereotactic arrhythmia radioablation for immunosuppressive refractory cardiac sarcoidosis and ventricular tachycardia

**DOI:** 10.3389/fcvm.2023.1213165

**Published:** 2023-07-21

**Authors:** Martijn H. van der Ree, Claudia Herrera Siklody, Mathieu Le Bloa, Patrizio Pascale, Alessandra P. Porretta, Cheryl C. Teres, Jorge Solana Munoz, Adrian Luca, Giulia Domenichini, Mahmut Ozasahin, Raphael Jumeau, Pieter G. Postema, Camillo Ribi, Jean Bourhis, Luis Schiappacasse, Etienne Pruvot

**Affiliations:** ^1^ Department of Cardiology, Lausanne University Hospital (CHUV), Lausanne, Switzerland; ^2^ Department of Cardiology, Amsterdam UMC Location University of Amsterdam, Amsterdam, Netherlands; ^3^ Heart Failure and Arrhythmias, Amsterdam Cardiovascular Sciences, Amsterdam, Netherlands; ^4^ Department of Radiation Oncology, Lausanne University Hospital (CHUV), Lausanne, Switzerland; ^5^ Division of Immunology and Allergy, Department of Medicine, Lausanne University Hospital (CHUV), Lausanne, Switzerland

**Keywords:** stereotactic arrhythmia radioablation (STAR), cardiac radioablation, ventricular tachycardia, cardiac sarcoidosis, radiotherapy, heart failure

## Abstract

**Background:**

Cardiac sarcoidosis is associated with heart failure, conduction abnormalities, and life-threatening arrhythmias including ventricular tachycardia (VT). Radiotherapy has been suggested as a treatment for extra-cardiac sarcoidosis in patients refractory to immunomodulatory treatment.

**Methods:**

The effectiveness and safety of low-dose whole-heart radiotherapy for therapy refractory cardiac sarcoidosis were evaluated in a pre- and post-intervention case report comparing the 54 months before and after treatment. Immunomodulatory low-dose whole-heart irradiation as sarcoidosis treatment consisted of a 2 × 2 Gy scheme. Additionally, high-dose single-fraction stereotactic arrhythmia radioablation of 1 × 20 Gy was applied to the pro-arrhythmic region to manage the ventricular tachycardia episodes. Cardiac sarcoidosis disease activity was measured by hypermetabolic areas on repeated fluorodeoxyglucose ([^18^F]FDG)-PET/computed tomography (CT) scans and by evaluating changes in ventricular tachycardia episodes before and after treatment.

**Results:**

One patient with therapy refractory progressive cardiac sarcoidosis and recurrent ventricular tachycardia was treated. The cardiac sarcoidosis disease activity showed a durable regression of inflammatory disease activity from 3 months onwards. The [^18^F]FDG-PET/CT scan at 54 months did not show any signs of active cardiac sarcoidosis, and a state of remission was achieved. The number of sustained VT episodes was reduced by 95%. We observed that the development of moderate aortic valve regurgitation was likely irradiation-related. No other irradiation-related adverse events occurred, and the left ventricular ejection fraction remained stable.

**Conclusion:**

We report here for the first time on the beneficial and lasting effects of combined immunomodulatory low-dose whole-heart radiotherapy and high-dose stereotactic arrhythmia radioablation in a patient with therapy refractory cardiac sarcoidosis and recurrent VT.

## Introduction

Sarcoidosis is a systemic inflammatory disease of unknown etiology (1). The disease is characterized by non-caseous granulomas and subsequent scarring in various organs including the heart. Cardiac sarcoidosis is associated with heart failure, conduction abnormalities, and life-threatening arrhythmias including ventricular tachycardia (VT) (2, 3). Treatment of cardiac sarcoidosis consists of immunomodulators and management of cardiac complications (4). In patients at risk of ventricular arrhythmias, an implantable cardioverter-defibrillator (ICD) is advised to prevent sudden cardiac death (5). However, despite immunosuppression, arrhythmic events occur frequently requiring anti-arrhythmic drugs (AAD) prescription and/or catheter ablations (5). In patients with AAD and catheter ablation refractory VT, stereotactic arrhythmia radioablation (STAR) has shown promising results; albeit, the worldwide experience is still very limited (6–10). Interestingly, since the 1980s, there are reports on the immunomodulatory effects of low-dose radiotherapy (11, 12). It has been suggested as an immunomodulatory treatment for patients with therapy refractory extra-cardiac sarcoidosis (13–15). Potentially, patients with both therapy refractory cardiac sarcoidosis and VT may benefit from a combined radiotherapy treatment. We report here on the beneficial and lasting effects of immunomodulatory low-dose whole-heart radiotherapy and high-dose STAR in a patient with cardiac sarcoidosis and recurrent VT refractory to conventional therapies.

## Case presentation

### Patient history and run-up to combined radiotherapy treatment

**Figure 1:**
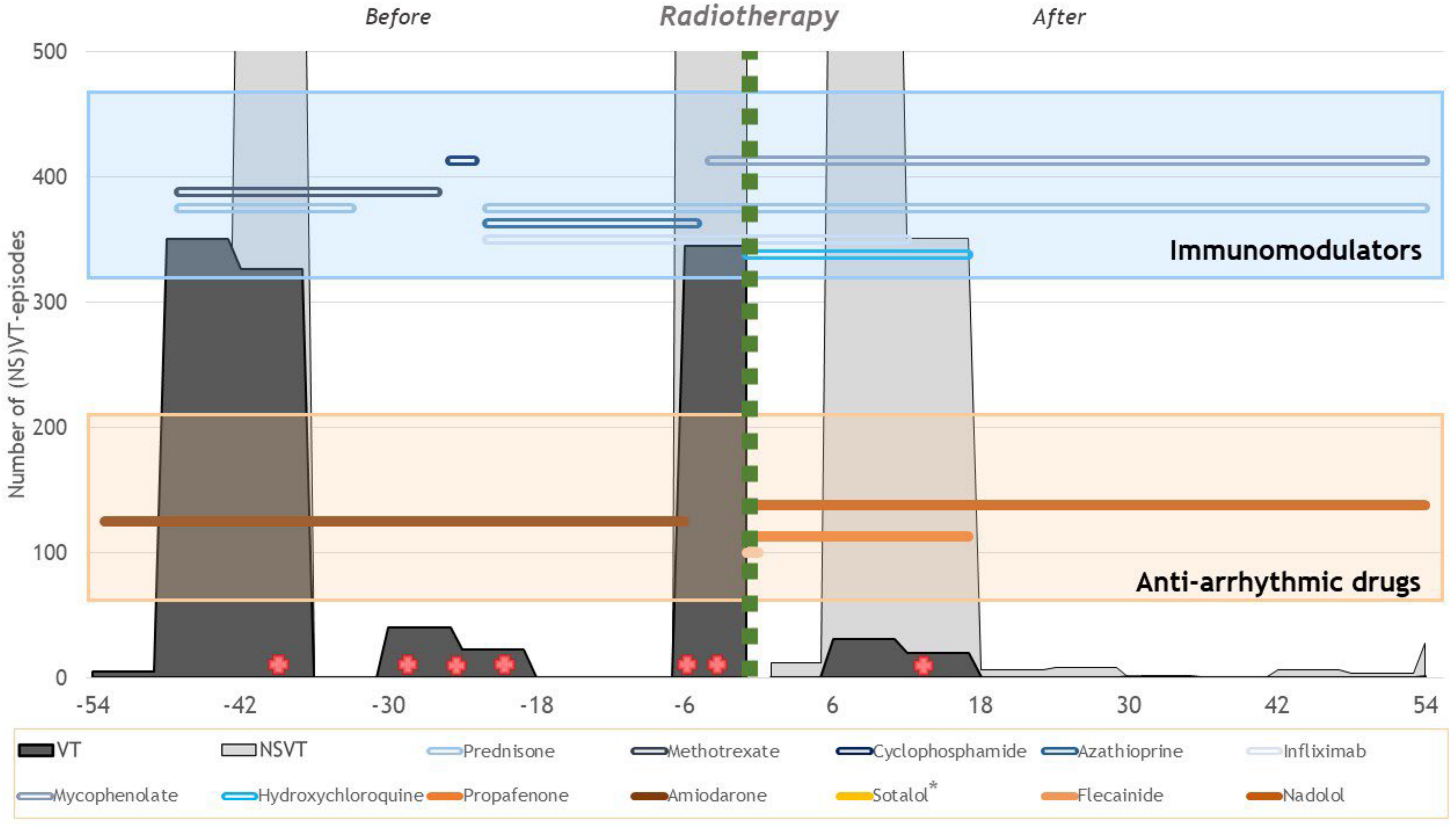
Overview of the ventricular arrhythmia burden and therapies before and after radiotherapy treatment. The *x*-axis shows the number of months relative to radiotherapy treatment. In gray, the number of (NS)VT episodes that occurred during the 6-month interval is presented. The green vertical line indicates the timing of the radiotherapy treatments. In the blue box, the lines show the use of the different immunomodulatory drugs over time. In the orange box, the lines show the use of the different anti-arrhythmic drugs over time. The red cross indicates invasive catheter ablations. *Sotalol has been used for less than a week.

At the age of 47 years, a female patient presented with dyspnea on exertion. The diagnostic work-up included ECG, Holter, echocardiography, computed tomography (CT) scan, cardiovascular magnetic resonance (CMR) scan, fluorodeoxyglucose ([^18^F]FDG)-PET/CT scan, and cardiac biopsy. The left ventricular ejection fraction was abnormal (49%). CMR revealed fibrotic lesions in the basal anterior, septal, and mid-anteroseptal segments of the left ventricle (LV). Hypermetabolic activity was also observed in these segments on the [^18^F]FDG-PET/CT scan as well as in the mid-inferior segment and right ventricle (RV). Endomyocardial biopsies were non-conclusive, which was interpreted as a failure to reach the target. Additionally, a third-degree atrioventricular block and complex premature ventricular contractions and VTs were present, and a cardiac resynchronization therapy with defibrillator (CRT-D) was implanted. No extra-cardiac signs of sarcoidosis were observed. The patient was diagnosed with isolated cardiac sarcoidosis based on the revised criteria of the Japanese guidelines (3). Immunomodulatory and AAD therapy was initiated and modified according to the clinical course (Figure 1).

One year after the diagnosis, the patient underwent a first catheter ablation because of recurrent VT episodes. Over the course of four years, several endo- and epicardial ablations were performed due to recurrent VT (Figure 1). The frequent VTs also resulted in mitral annular dilation and regurgitation. Despite immunomodulatory treatment, repeated [^18^F]FDG-PET/CT scans persistently showed focal hypermetabolic activity in the LV indicating an active disease. Consequently, heart transplantation was considered. At 3.4 years after the initial presentation and after already four catheter ablations, the patient developed an electrical storm as a consequence of an amiodarone-induced thyrotoxicosis. Amiodarone was stopped and the corticosteroid scheme was altered. The electrical storm was managed by a fifth catheter ablation targeting the RV septum. Five months later, the patient was again admitted for an electrical storm with VTs originating from both the LV and RV, and a sixth RV catheter ablation was performed. A basal anterior floating LV thrombus precluded a transseptal LV catheter ablation, and she was therefore considered for the compassionate use of experimental immunomodulatory low-dose whole-heart radiotherapy and STAR due to combined therapy refractory disease. No bipolar or alcohol ablation was performed. The ethics committee approved the use of the radiotherapy treatment.

### STAR planning and delivery

A 4D planning CT was performed for both radiotherapy treatments. Image registration and delineations were performed using the Velocity AI software (Varian Medical Systems Inc., Palo Alto, CA, United States). Organs at risk (OAR), including the lungs, bronchial tree, esophagus, and stomach, were delineated. To spare OAR, inverse planning was used by applying standard dose constraints (16).

For the immunomodulatory low-dose whole-heart irradiation, the entire heart was delineated by the radiation oncologist as clinical target volume (CTV). An internal target volume (ITV) was created with additional margins to compensate for cardiorespiratory motion based on the 4D-CT. Isotropic expansion of the ITV was performed to generate the planning target volume (PTV). The immunomodulatory radiotherapy consisted of a 2 × 2 Gy scheme prescribed to the whole-heart PTV. The treatment plan was computed using TomoHDA System Planning Station version 5.1.1.6 (Accuray Inc., Madison, WI, United States). The treatment was delivered by a TomoTherapy HDA Treatment System (Accuray Inc., Sunnyvale, CA, United States).

The STAR pro-arrhythmic substrate (VT-CTV) was determined and delineated by the electrophysiologist and radiation oncologist using previously acquired electroanatomical maps and imaging modalities including CMR. From this VT-CTV, a VT-PTV was generated by adding a 3 mm uncertainty margin. To this VT-PTV, a dose of 20 Gy was prescribed to the 85% isodose to manage the arrhythmias. The treatment plan was computed using MultiPlan version 5.3.0 treatment planning system (Accuray Inc., Sunnyvale, CA, United States). STAR treatment was delivered using the CyberKnife (Accuray Inc., Sunnyvale, CA, United States) treatment system. The RV ICD lead was used as a fiducial marker for real-time tracking of the motion. It was decided to prescribe 20 Gy instead of the conventional 25 Gy because the dose of the low-dose whole-heart treatment was considered in the total dose.

The two treatments were performed within the same week.

## Results

The CTV and PTV for the immunomodulatory radiotherapy were 688 and 1,162 cc, respectively. For the STAR treatment, the VT-CTV consisted of the basal anterior and basal anteroseptal segments resulting in a VT-CTV of 8 cc and a VT-PTV of 16 cc. The mean irradiation dose (Dmean) to the heart for the two treatments combined was 9.1 Gy, and the maximum dose (Dmax) received was 27.5 Gy.

**Figure 2:**
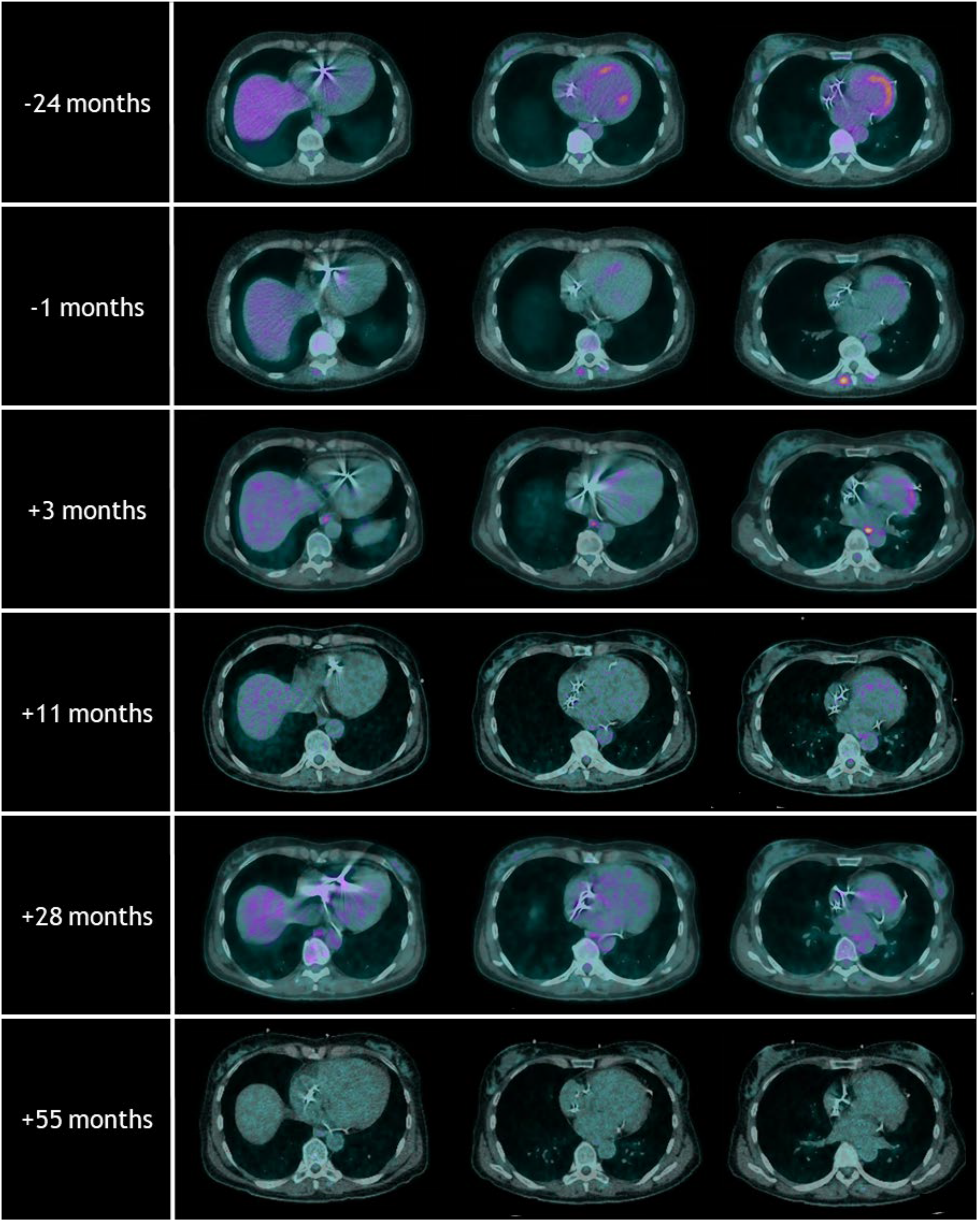
Overview of the repeated [^18^F]FDG-PET/CT scans with the timing relative to radiotherapy treatment. Note the hypermetabolic areas of the anteroseptal segments at the [^18^F]FDG-PET/CT scans prior to and 24 months before radiotherapy. Follow-up [^18^F]FDG-PET/CT scans after radiotherapy showed regression of these local hypermetabolic areas. The [^18^F]FDG-PET/CT scan at 55 months did not show any signs of active cardiac sarcoidosis.

Figure 2 shows an overview of the repeated [^18^F]FDG-PET/CT scans. On the [^18^F]FDG-PET/CT scans prior to and 2 years before treatment, hypermetabolic areas of the anteroseptal segment and the posterior papillary muscle were observed. Three months after treatment, the hypermetabolic cardiac activity was reduced. Only in the basal anterior segment, corresponding to VT-PTV, a mild increase in metabolic activity was observed. Another [^18^F]FDG-PET/CT scan was performed 11 months after treatment and showed regression of this local hypermetabolic activity and no new pathological activity. The [^18^F]FDG-PET/CT scan at 55 months did not show any signs of active cardiac sarcoidosis, and a state of remission was achieved.

Figure 1 shows an overview of the ventricular arrhythmia burden and different therapies. Comparing the 54 months before and after radiotherapy treatments, the number of sustained VT episodes was reduced by 95% (1,089 pre-treatment vs. 53 after treatment). The number of non-sustained VT episodes was also reduced from 3,051 pre-treatment to 1,289 after treatment (−58%). It is important to acknowledge that this reduction in VT episodes is not only attributable to the radiotherapy treatments as several catheter ablations have been performed (Figure 1, red cross). Seven months after treatment, a new electrical storm occurred in the context of an influenza infection which resolved spontaneously. Because of the multiple arrhythmia recurrences after STAR, eventually, a seventh successful catheter ablation was performed 16 months after radiotherapy treatment. The recurrence was located on the border of the VT-CTV at the basal anteroseptal and mid-anteroseptal segments. Afterward, only two sustained VT episodes treated by ATP occurred during the following 38 months. The mitral valve regurgitation significantly reduced in the months after treatment.

During the follow-up of 55 months, we observed that the development of moderate aortic valve regurgitation was likely irradiation-related. No other irradiation-related adverse events occurred, and the left ventricular ejection fraction remained stable.

## Discussion

We present a patient with therapy refractory cardiac sarcoidosis and VTs treated with low-dose whole-heart radiotherapy and high-dose STAR. To our knowledge, this is the first report on the use of low-dose whole-heart radiotherapy as an immunomodulatory treatment for cardiac sarcoidosis. We showed a reduction in both inflammatory disease activity and VT episodes.

Previously, radiotherapy in cardiology was mostly known for its late radiation-induced cardiovascular complications (17). A paradigm shift occurred when STAR was introduced as a therapeutic option for therapy refractory ventricular arrhythmias (6–10). This shift also occurred in our tertiary academic referral center as the previous experiences with STAR were favorable (9).

The low-dose whole-heart irradiation was performed as a compassionate and experimental immunomodulatory treatment of cardiac sarcoidosis. The decision to perform this treatment was based on previous reports on the immunomodulatory effects of radiotherapy and the concurrent risks of current and future treatments (e.g., VT storm, complications of heart transplantation) (11, 12, 18). Reports on the treatment of patients with extra-cardiac sarcoidosis, mainly neurosarcoidosis, showed that radiotherapy could indeed be beneficial in managing disease activity (13–15). Reported doses widely varied between 10 and 60 Gy divided over 1–15 fractions. For safety reasons, we deviated from those reports and prescribed a lower dose. The disease activity was monitored by repeated [^18^F]FDG-PET/CT scans and showed a durable regression of inflammatory disease activity from 3 months onwards. It is important to mention that the dose received by the heart due to the combined radiotherapy treatment was higher than the initially prescribed, i.e., 4 Gy (Dmean 9.1 Gy). During follow-up, the only irradiation-related adverse event we observed was the development of moderate aortic valve disease at 46 months that is being conservatively managed (Dmean 24.7 Gy, Dmax 26.8 Gy). We consider this a mild and manageable adverse event, especially in light of complications of advancing disease and ultimately heart transplantation (4, 18).

The STAR treatment was performed because of its contraindication to catheter ablation. The reported efficacy and safety of STAR are promising in both ischemic and non-ischemic cardiomyopathies (6–10). European efforts are being undertaken to clinically validate STAR (STOPSTORM.eu project). In this patient, we were able to achieve a remarkable reduction in VTs. Ventricular arrhythmias in cardiac sarcoidosis are most often caused by reentry around post-inflammatory myocardial scars but may also occur as a consequence of active inflammation (2). Whether the marked reduction in VTs complementary to the performed catheter ablations was induced by the immunomodulatory effects of low-dose whole-heart irradiation, the anti-arrhythmic effect of high-dose STAR, a combination, or is due to other factors (e.g., natural course of the disease, medication) remains to be elucidated. Targeting the arrhythmogenic substrate for STAR in a patient with cardiac sarcoidosis is challenging as the substrate is often extensive and complex and may alter over time as the disease progresses (2). For this patient, the extensive information from the electroanatomical maps guided the delineation. The use of this targeting method in combination with the use of the system, which can track respiratory motion in real time during treatment delivery, allowed for a small target volume. Treating small volumes is especially important in this non-ischemic patient category when considering redo-STAR and that the underlying disease may progress (19, 20).

## Conclusion

Here, we report for the first time on the beneficial and lasting effects of combined experimental immunomodulatory low-dose whole-heart radiotherapy and high-dose STAR in a patient with therapy refractory cardiac sarcoidosis and recurrent VT. Based on these promising results, radiotherapy could be considered in a compassionate and experimental manner for selected therapy refractory patients. However, caution is advised regarding (extra-)cardiac complications.

## Data Availability

The raw data supporting the conclusions of this article will be made available by the authors, without undue reservation.
